# Divergent ectopic fat distribution and adipokine/hepatokine levels characterize the metabolically healthy overweight/obesity phenotype

**DOI:** 10.1016/j.isci.2026.117672

**Published:** 2026-09-30

**Authors:** Antonio Clavero-Jimeno, Juan J. Martin-Olmedo, Alessandro Gatti, Adrián Cortés-Martín, Alba Camacho-Cardenosa, Lucas Jurado-Fasoli, Manuel Dote-Montero, Marcos Molina-Fernandez, Alejandro López-Vázquez, Amaya Lopez-Pascual, Matías A. Ávila, Estrella Petrina, Ana Zugasti, Iñigo Urdiain, Mara Concepción, Rafael Cabeza, José L. Martín-Rodríguez, Idoia Labayen, Jonatan R. Ruiz

**Affiliations:** 1 Department of Physical Education and Sports, Faculty of Sport Sciences, Sport and Health University Research Institute (iMUDS), University of Granada, Granada, Spain; 2 Department of Physiology, Faculty of Medicine, Sport and Health University Research Institute (iMUDS), University of Granada, Granada, Spain; 3 Novo Nordisk Foundation Center for Basic Metabolic Research, University of Copenhagen, Copenhagen, Denmark; 4 Laboratory of Adapted Motor Activity, Department of Public Health, Experimental Medicine and Forensic Science, University of Pavia, Pavia, Italy; 5 National PhD Programme in One Health Approaches to Infectious Diseases and Life Science Research, Department of Public Health, Experimental and Forensic Medicine, University of Pavia, Pavia, Italy; 6 Instituto de Investigación Biosanitaria de Granada (ibs. GRANADA), Granada, Spain; 7 Centro de Investigación Biomédica en Red: Fisiopatología de la Obesidad y Nutrición (CIBEROBN), Instituto de Salud Carlos III, Madrid, Spain; 8 Obesity and Diabetes Clinical Research Section, Phoenix Epidemiology and Clinical Research Branch, National Institute of Diabetes and Digestive and Kidney Diseases, National Institutes of Health, Phoenix, AZ, USA; 9 Hepatology Laboratory, Solid Tumors Program, CIMA, CCUN, University of Navarra, Pamplona, Spain; 10 Navarra Institute for Health Research (IdiSNA), Pamplona, Spain; 11 CIBERehd, Instituto de Salud Carlos III, Madrid, Spain; 12 Servicio de Endocrinologia y Nutricion, Hospital Universitario de Navarra, Pamplona, Spain; 13 ELIKOS Research Group, Institute for Sustainability & Food Chain Innovation, Department of Health Sciences, Public University of Navarre, Pamplona, Spain; 14 Department of Electrical, Electronic and Communications Engineering, Public University of Navarre, Pamplona, Spain; 15 Radiology Department, University Hospital Clinic San Cecilio, Granada, Spain

**Keywords:** ectopic fat, hepatic steatosis, pancreatic steatosis, cytokines, obesity phenotypes, gut microbiome

## Abstract

The metabolically healthy overweight/obesity (MHOO) phenotype combines excess adiposity with preserved cardiometabolic health. We compared specific fat depots, circulating adipokine and hepatokine levels, and fecal microbiota composition between 384 adults classified as MHOO (*n* = 99) or metabolically unhealthy overweight/obesity (MUOO; *n* = 285). Magnetic resonance imaging was used to quantify visceral adipose tissue (VAT) volume, hepatic and pancreatic fat fractions, abdominal and mid-thigh intermuscular adipose tissue (IMAT), and subcutaneous adipose tissue (SAT). Despite similar whole-body fat mass, MHOO showed lower VAT volume, hepatic and pancreatic fat fractions, abdominal IMAT volume, abdominal VAT-to-SAT ratio, and mid-thigh IMAT area, together with higher mid-thigh SAT area. MHOO also had higher adiponectin and sex hormone-binding globulin (SHBG) and lower retinol-binding protein 4 (RBP4) levels. Fecal microbiota composition was similar between groups. VAT volume and hepatic fat fraction were the strongest discriminative features, supporting fat distribution rather than total adiposity as a key feature of preserved metabolic health.

## Highlights

• MHOO shows lower visceral, hepatic, and pancreatic fat and higher mid-thigh subcutaneous fat
• Selected adipokines and hepatokines differ between MHOO and MUOO
• Fecal microbiota composition is similar between MHOO and MUOO
• Visceral and hepatic fat are major distinguishing factors between MHOO and MUOO

## Graphical abstract

**Figure:**
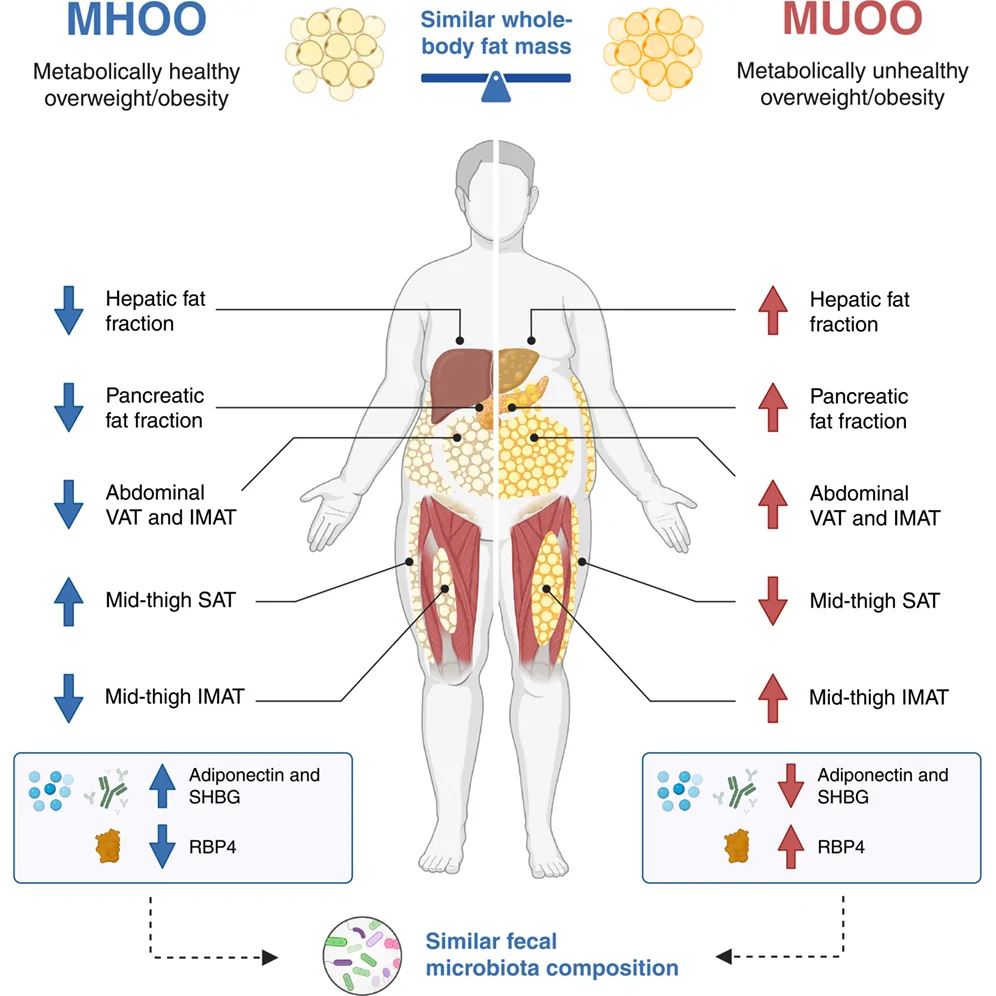

## Introduction

Overweight and obesity are major global public health concerns and are well-established risk factors for cardiometabolic diseases.^1^^,^^2^ However, the relationship between adiposity and metabolic dysfunction is nonlinear. A distinct subset of individuals with overweight or obesity displays a paradoxical resilience to insulin resistance, dyslipidemia, and hypertension,^3^^,^^4^^,^^5^ the so-called metabolically healthy overweight/obesity (MHOO).^3^^,^^6^ While often dismissed as a transient state,^7^^,^^8^ the MHOO phenotype offers a unique human model to identify the protective mechanisms that decouple pathological fat accumulation from systemic cardiometabolic health. Elucidating the biological factors that sustain this equilibrium is critical, as it may reveal the mechanisms that govern the progression toward a metabolically unhealthy overweight/obesity (MUOO) phenotype, with profound implications for preclinical risk identification and personalized obesity management.^9^

Despite the presence of excess whole-body fat mass in both MHOO and MUOO, differences in fat distribution appear to be determinants of their different clinical trajectories.^5^^,^^6^^,^^10^ Beyond the established risks associated with visceral adipose tissue (VAT) and hepatic steatosis, cardiometabolic health is likely governed by more complex distribution of fat across specific depots.^4^^,^^11^^,^^12^ Emerging evidence suggests that fat accumulation within the pancreas and intermuscular adipose tissue (IMAT) may act as potent drivers of cardiometabolic dysfunction.^13^^,^^14^ Yet, the specific distribution patterns of these relatively understudied depots, particularly the differences between abdominal and mid-thigh IMAT,^15^ remain poorly defined in the context of metabolic health, as does the role of pancreatic fat in populations without type 2 diabetes.^16^

These depot-specific differences in adiposity may drive distinct patterns of circulating signaling molecules across obesity phenotypes,^6^ including adipokines and hepatokines, whose dysregulation is linked to ectopic lipid accumulation, chronic inflammation, and insulin resistance.^17^^,^^18^ Moreover, gut microbiota dysbiosis has been implicated in metabolic dysfunction in obesity,^19^ pointing to a potential mechanistic link between host-microbiota interactions and the progression from health to disease.^20^ Despite these known connections, current research remains largely fragmented. Many studies are limited by small sample sizes,^21^^,^^22^ unbalanced sex distributions,^20^^,^^21^^,^^22^^,^^23^^,^^24^^,^^25^ or the isolated assessment of these features, rather than their integrated evaluation within the same individuals.^26^

In this study, we used an integrative deep-phenotyping approach in adults with overweight/obesity to characterize differences between the MHOO and MUOO phenotypes. Specifically, we combined magnetic resonance imaging (MRI) quantification of multiple fat depots with circulating adipokine/hepatokine profiling and fecal microbiota sequencing to identify the features associated with the MHOO phenotype.

## Results

### Deep phenotyping reveals a favorable cardiometabolic fingerprint in MHOO

The descriptive characteristics of the participants are shown in Table 1. Compared with MUOO, participants with MHOO exhibited lower neck and waist circumferences, waist-to-hip ratio, fat-free mass, and mid-thigh skeletal muscle tissue (all *p* values < 0.001; Table 1). Moreover, participants with MHOO showed a more favorable cardiometabolic profile than those with MUOO, including improved glucose homeostasis (i.e., lower insulin, homeostasis model of assessment for insulin resistance [HOMA-IR], glycated hemoglobin [HbA1c], and mean glucose levels across 24 h, daytime, and nighttime, as well as reduced coefficient of variations [CVs] of glucose over 24 h and daytime; all *p* values ≤ 0.043; Table 1), a healthier blood lipid profile (i.e., higher apolipoprotein A1 and lower apolipoprotein B; both *p* values < 0.001; Table 1), and lower levels of the liver enzymes alanine aminotransferase (ALT) and γ-glutamyltransferase (GGT) (both *p* values < 0.001; Table 1). However, no significant differences between the MHOO and MUOO phenotypes were observed for hip circumference, fat mass, nocturnal CV glucose, total cholesterol, low-density lipoprotein cholesterol, alkaline phosphatase, sedentary time, light physical activity (LPA), moderate-to-vigorous physical activity (MVPA), or energy, carbohydrate, fat, protein intake, or Mediterranean Diet Adherence Screener (MEDAS) score (all *p* values ≥ 0.072; Table 1).

**Table 1:** Descriptive characteristics of participants with MHOO and MUOO

	–	All (*n* = 384)	–	MHOO (*n* = 99)	–	MUOO (*n* = 285)	–
	*N*	–	*n*	–	*n*	–	*p* value
Age	384	47.6 (7.7)	99	45.9 (7.7)	285	48.1 (7.7)	**0.005**
Men, *n* (%)	–	193 (50.3)	–	42 (42.4)	–	151 (53.0)	0.090

Women, *n* (%)	–	191 (49.7)	–	57 (57.6)	–	134 (47.0)	–

**Anthropometry and body composition**							

Body weight (kg)	384	96.9 (14.7)	99	93.5 (15.3)	285	98.1 (14.3)	**<0.001**
Height (cm)	384	169.0 (9.4)	99	168.3 (9.7)	285	169.2 (9.3)	0.268
Body mass index (kg/m^2^)	384	33.9 (3.7)	99	32.9 (3.5)	285	34.2 (3.7)	**0.009**
Overweight, *n* (%)	–	54 (14.1)	–	17 (17.0)	–	37 (13.0)	0.387
Obesity, *n* (%)	–	330 (85.9)	–	82 (82.8)	–	248 (87.0)	–
Neck circumference (cm)	384	39.7 (4.0)	99	38.1 (3.9)	285	40.3 (3.9)	**<0.001**
Waist circumference (cm)	384	104.4 (10.8)	99	99.7 (10.8)	285	106.1 (10.4)	**<0.001**
Hip circumference (cm)	384	116.6 (9.1)	99	116.2 (8.2)	285	116.8 (9.4)	0.696
Waist-to-hip ratio	384	0.90 (0.09)	99	0.86 (0.10)	285	0.91 (0.09)	**<0.001**
Fat-free mass (kg)	384	58.0 (11.6)	99	55.6 (12.2)	285	58.8 (11.3)	**<0.001**
Fat mass (kg)	384	38.1 (8.3)	99	37.2 (8.2)	285	38.4 (8.3)	0.077
Fat mass (%)	384	39.4 (6.9)	99	40.0 (7.0)	285	39.3 (6.8)	0.236
Mid-thigh skeletal muscle tissue (cm^2^)	349	135 (31)	86	129 (30)	263	137 (31)	**<0.001**

**Blood pressure**							

Systolic blood pressure (mmHg)	382	121 (14)	98	112 (9)	284	124 (14)	**<0.001**
Diastolic blood pressure (mmHg)	382	82 (10)	98	74 (6)	284	85 (9)	**<0.001**

**Glucose homeostasis**							

Fasting glucose (mg/dL)	384	92 (11)	99	88 (7)	285	93 (12)	**0.003**
Fasting insulin (mU/L)	383	11.3 (5.9)	99	9.1 (4.3)	284	12.0 (6.2)	**<0.001**
HOMA-IR	382	2.61 (1.49)	98	2.01 (1.02)	284	2.82 (1.57)	**<0.001**
HbA1c (%)	384	5.4 (0.4)	99	5.3 (0.3)	285	5.5 (0.4)	**0.020**
24-h mean glucose (mg/dL)	326	102 (11)	85	99 (9)	241	104 (11)	**<0.001**
Diurnal mean glucose (mg/dL)	326	105 (11)	85	101 (9)	241	106 (11)	**0.001**
Nocturnal mean glucose (mg/dL)	326	98 (12)	85	93 (10)	241	99 (12)	**<0.001**
24-h CV glucose (%)	326	14.5 (3.5)	85	13.7 (3.6)	241	14.8 (3.6)	**0.043**
Diurnal CV glucose (%)	326	14.5 (3.7)	85	13.6 (3.6)	241	14.8 (3.7)	**0.020**
Nocturnal CV glucose (%)	326	9.3 (2.8)	85	8.9 (2.8)	241	9.5 (2.8)	0.072

**Blood lipid profile**							

Total cholesterol (mg/dL)	384	208 (36)	99	207 (38)	285	208 (36)	0.735
HDL-C (mg/dL)	384	53 (12)	99	60 (11)	285	51 (11)	**<0.001**
LDL-C (mg/dL)	384	132 (31)	99	130 (33)	285	133 (31)	0.673
Triglycerides (mg/dL)	384	121 (64)	99	87 (28)	285	133 (68)	**<0.001**
Apolipoprotein A1 (mg/dL)	384	163 (31)	99	176 (26)	285	158 (31)	**<0.001**
Apolipoprotein B (mg/dL)	384	104 (23)	99	95 (20)	285	107 (23)	**<0.001**

**Liver enzymes**							

Alanine aminotransferase (U/L)	384	30 (23)	99	25 (14)	285	32 (26)	**<0.001**
γ-Glutamyltransferase (U/L)	384	35 (42)	99	25 (15)	285	39 (48)	**<0.001**
Alkaline phosphatase (U/L)	384	71 (20)	99	70 (18)	285	72 (20)	0.962

**Sedentary time and physical activity**							

Sedentary time (min/day)	326	661.8 (83.0)	85	655.4 (90.0)	241	664.1 (80.7)	1.000
LPA (min/day)	326	246.9 (58.0)	85	245.1 (61.7)	241	247.6 (56.8)	1.000
MVPA (min/day)	326	24.8 (21.7)	85	25.3 (22.3)	241	24.7 (21.5)	1.000

**Dietary intake**							

Energy intake (kcal/day)	384	1,941 (473)	99	1,904 (469)	285	1,954 (474)	1.000
Carbohydrate intake (% EI)	384	38 (7)	99	38 (8)	285	38 (7)	1.000
Fat intake (% EI)	384	41 (7)	99	41 (8)	285	41 (7)	1.000
Protein intake (% EI)	384	18 (3)	99	18 (3)	285	18 (3)	0.984
MEDAS score	382	7.1 (1.8)	99	7.3 (1.8)	283	7.0 (1.8)	1.000

**Medications**							

Antihypertensives, *n* (%)	–	65 (16.9)	–	0 (0)	–	65 (22.8)	–
Lipid-lowering, *n* (%)	–	35 (9.1)	–	0 (0)	–	35 (12.3)	–

Data are presented as mean (standard deviation) unless otherwise stated. Bold *p* values denote statistically significant differences (*p* < 0.05). Two-sided *p* values are derived from analysis of covariance (parametric or non-parametric, depending on data distribution) adjusted for age, sex, and body mass index, comparing MHOO and MUOO for continuous variables, and from chi-square tests for categorical variables. CV, coefficient of variation; EI, energy intake; HbA1c, glycated hemoglobin; HDL-C, high-density lipoprotein cholesterol; HOMA-IR, homeostasis model assessment of insulin resistance; LDL-C, low-density lipoprotein cholesterol; LPA, light physical activity; MEDAS, Mediterranean Diet Adherence Screener; MVPA, moderate-to-vigorous physical activity.

### Ectopic fat partitioning distinguishes MHOO from MUOO

Participants with MHOO had significantly lower VAT volume (mean difference: −411 cm^3^; 95% confidence interval [CI], −535 to −288; *p* < 0.001; Cohen’s d = 0.750; Figure 1A; Table 2), hepatic fat fraction (mean difference: −4.0%; 95% CI, −5.2 to −2.7; *p* < 0.001; Cohen’s d = 0.645), and pancreatic fat fraction (mean difference: −1.5%; 95% CI, −3.1 to 0.0; *p* = 0.004; Cohen’s d = 0.225) compared with MUOO (Figures 1B and 1C; Table 2).

**Figure 1:**
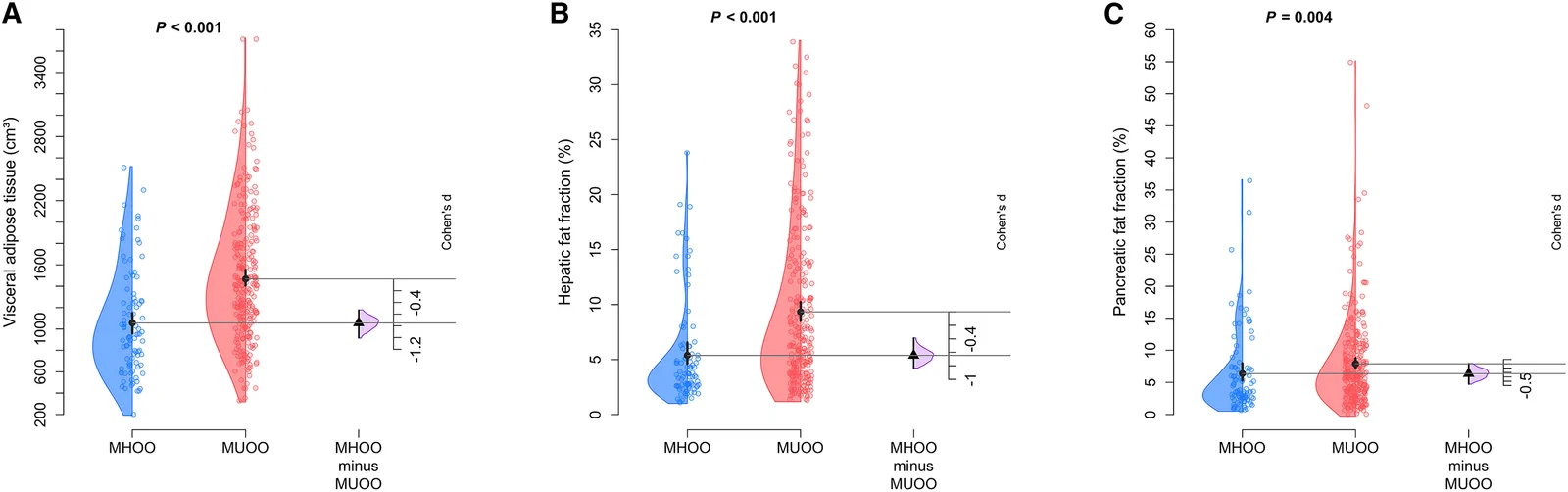
Differences between MHOO and MUOO in VAT, hepatic fat fraction, and pancreatic fat fraction (A) Visceral adipose tissue. (B) Hepatic fat fraction. (C) Pancreatic fat fraction. Data are displayed using Gardner-Altman plots as individual raw values and group means with 95% confidence intervals, together with the standardized effect size (Cohen’s d) and its 95% bootstrap confidence interval. Two-sided *p* values from analysis of covariance (parametric or non-parametric depending on data distribution) evaluating the differences between groups after adjusting for age, sex, and whole-body fat mass. MHOO, metabolically healthy overweight/obesity; MUOO, metabolically unhealthy overweight/obesity.

**Table 2:** Differences in specific abdominal and mid-thigh adipose tissue depots, and organ-specific fat deposition between participants with MHOO and MUOO

	MHOO		MUOO		Difference between groups (MHOO - MUOO)		
	*n*	Mean (95% CI)	*n*	Mean (95% CI)	Mean difference (95% CI)	*p* value	Cohen’s d
**Abdominal adipose tissue depots**							

Visceral adipose tissue (cm^3^)	92	1,056 (959, 1,154)	272	1,468 (1,398, 1,538)	−411 (−535, −288)	**<0.001**	0.750
Intermuscular adipose tissue (cm^3^)	92	329 (301, 356)	272	373 (354, 392)	−44 (−78, −11)	**0.044**	0.297
Subcutaneous adipose tissue (cm^3^)	92	2,964 (2,793, 3,136)	272	2,961 (2,858, 3,064)	3 (−201, 208)	0.928	0.004
VAT-to-SAT ratio	92	0.38 (0.34, 0.43)	272	0.55 (0.52, 0.59)	−0.17 (−0.22, −0.11)	**<0.001**	0.643

**Mid-thigh adipose tissue depots**							

Intermuscular adipose tissue (cm^2^)	86	27 (26, 29)	263	31 (30, 32)	−4 (−6, −2)	**<0.001**	0.502
Subcutaneous adipose tissue (cm^2^)	86	140 (129, 151)	263	119 (113, 126)	21 (8, 35)	**<0.001**	0.377

**Organ-specific fat deposition**							

Hepatic fat fraction (%)	95	7.1 (6.2, 8.0)	273	10.9 (10.1, 11.7)	−4.0 (−5.2, −2.7)	**<0.001**	0.645
Pancreatic fat fraction (%)	96	6.4 (5.1, 7.7)	275	7.9 (7.1, 8.7)	−1.5 (−3.1, 0.0)	**0.004**	0.225

Data are presented as descriptive means with 95% confidence intervals and as unadjusted raw mean differences (MHOO minus MUOO) with 95% confidence intervals. Bold *p* values denote statistically significant differences (*p* < 0.05). Two-sided *p* values are derived from adjusted analyses of covariance (parametric or non-parametric, as appropriate) including sex, age, and whole-body fat mass as covariates. Therefore, the confidence intervals for raw mean differences are descriptive and are not expected to correspond directly to the reported adjusted *p* values. SAT, subcutaneous adipose tissue; VAT, visceral adipose tissue.

Moreover, participants with MHOO showed lower abdominal IMAT volume (mean difference: −44 cm^3^; 95% CI, −78 to −11; *p* = 0.044; Cohen’s d = 0.297; Figure 2A; Table 2) and abdominal VAT-to-subcutaneous adipose tissue (SAT) ratio (mean difference: −0.17; 95% CI, −0.22 to −0.11; *p* < 0.001; Cohen’s d = 0.643; Figure 2B; Table 2) compared with MUOO. No significant between-group differences were observed in abdominal SAT volume (*p* = 0.928; Table 2).

**Figure 2:**
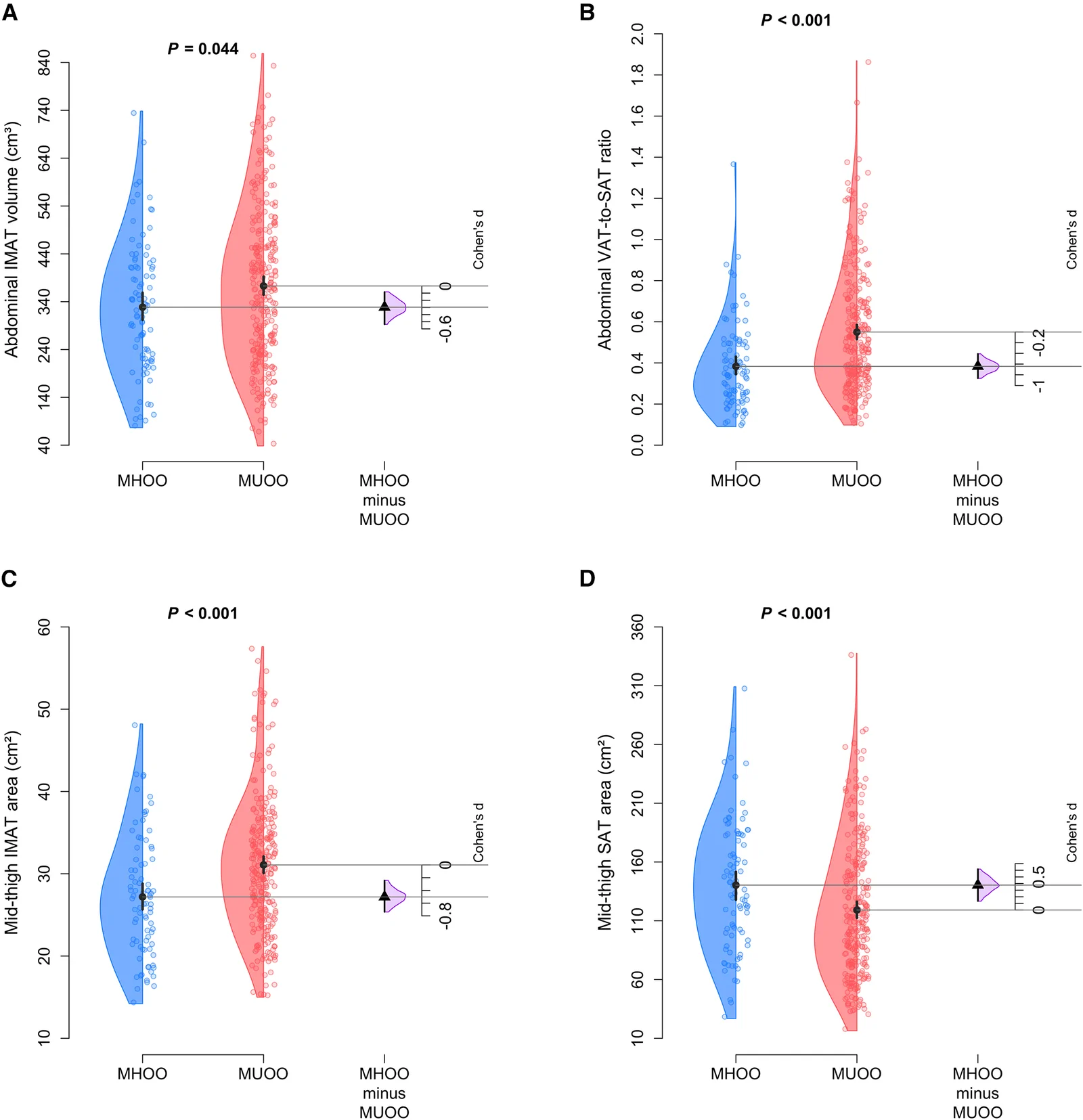
Differences between MHOO and MUOO in abdominal IMAT, abdominal VAT-to-SAT ratio, mid-thigh IMAT area, and mid-thigh SAT area (A) Abdominal intermuscular adipose tissue (IMAT) volume. (B) Abdominal visceral adipose tissue (VAT) to subcutaneous adipose tissue (SAT) ratio. (C) Mid-thigh IMAT area. (D) Mid-thigh SAT area. Data are displayed using Gardner-Altman plots as individual raw values and group means with 95% confidence intervals, together with the standardized effect size (Cohen’s d) and its 95% bootstrap confidence interval. Two-sided *p* values are derived from analysis of covariance (parametric or non-parametric depending on data distribution) evaluating the differences between groups after adjusting for age, sex, and whole-body fat mass. Abdominal VAT-to-SAT ratio was assessed by computing the ratio between VAT volume and abdominal SAT volume. MHOO, metabolically healthy overweight/obesity; MUOO, metabolically unhealthy overweight/obesity.

At the mid-thigh level, participants with MHOO also showed a lower IMAT area (mean difference: −4 cm^2^; 95% CI, −6 to −2; *p* < 0.001; Cohen’s d = 0.502; Figure 2C; Table 2) and a higher SAT area (mean difference: 21 cm^2^; 95% CI, 8 to 35; *p* < 0.001; Cohen’s d = 0.377) compared with MUOO (Figure 2D; Table 2).

We also conducted sex-stratified analyses of specific fat depots. Overall, the results were similar and consistent with the main analyses. However, a lower pancreatic fat fraction was observed in women with MHOO than in women with MUOO (mean difference: −2.4%; 95% CI, −4.0 to −0.8; *p* = 0.031; Cohen’s d = 0.419; Table S1), whereas this difference was not observed in men.

### The MHOO phenotype is linked to distinct levels of selected circulating adipokines and hepatokines

Participants with MHOO had higher circulating levels of the adipokine adiponectin (mean difference: 2.3 μg/mL; 95% CI, 0.6 to 4.0; *p* = 0.043; Cohen’s d = 0.466) and lower retinol-binding protein 4 (RBP4) levels (mean difference: −8.9 μg/mL; 95% CI, −13.5 to −4.2; *p* = 0.004; Cohen’s d = 0.577) compared with MUOO (Table 3). No significant differences were observed for dipeptidyl peptidase-4 (DPP4), interleukin-6 (IL-6), leptin, omentin, or resistin (all *p* values ≥ 0.913; Table 3).

**Table 3:** Differences in adipokines and hepatokines between participants with MHOO and MUOO

	MHOO		MUOO		Difference between groups (MHOO − MUOO)		
	*n*	Mean (95% CI)	*n*	Mean (95% CI)	Mean difference (95% CI)	*p* value	Cohen’s d
**Adipokines**							

Adiponectin (μg/mL)	48	8.7 (7.6, 9.8)	143	6.4 (5.9, 6.9)	2.3 (0.6, 4.0)	**0.043**	0.466
DPP4 (μg/mL)	48	0.98 (0.93, 1.03)	143	1.03 (0.99, 1.07)	−0.05 (−0.15, 0.04)	0.935	0.170
Interleukin-6 (pg/mL)	44	2.2 (2.0, 2.5)	142	2.5 (2.3, 2.7)	−0.3 (−0.7, 0.2)	0.913	0.170
Leptin (μg/L)	48	59.8 (53.8, 65.8)	137	62.7 (58.2, 67.2)	−2.9 (−13.6, 7.8)	0.994	0.085
Omentin (ng/mL)	48	58.4 (52.9, 63.9)	142	60.4 (52.9, 67.9)	−2.0 (−15.2, 11.2)	0.935	0.041
Resistin (ng/mL)	48	4.1 (3.7, 4.5)	144	4.7 (4.4, 5.1)	−0.6 (−1.4, 0.2)	0.935	0.226
RBP4 (μg/mL)	48	29.2 (26.7, 31.7)	142	38.1 (36.0, 40.1)	−8.9 (−13.5, −4.2)	**0.004**	0.577

**Hepatokines**							

ANGPTL3 (ng/mL)	48	443.4 (410.6, 476.2)	142	422.9 (405.5, 440.3)	20.5 (−32.1, 73.1)	1.000	0.131
ANGPTL4 (ng/mL)	46	0.89 (0.62, 1.16)	143	0.86 (0.67, 1.05)	0.03 (−0.45, 0.51)	1.000	0.020
β-Klotho (ng/mL)	48	11.0 (10.6, 11.4)	142	11.7 (10.9, 12.5)	−0.7 (−2.0, 0.5)	1.000	0.143
Clusterin (μg/mL)	48	232.1 (221.5, 242.6)	142	231.7 (226.4, 237.1)	0.3 (−16.4, 17.0)	1.000	0.007
Follistatin (ng/mL)	46	0.88 (0.83, 0.93)	143	1.03 (0.98, 1.08)	−0.15 (−0.25, −0.05)	0.149	0.431
GDF15 (ng/mL)	47	0.27 (0.25, 0.29)	144	0.28 (0.27, 0.29)	−0.01 (−0.04, 0.02)	1.000	0.090
SHBG (nmol/L)	45	27.5 (24.0, 31.0)	140	21.8 (19.8, 23.7)	5.7 (−0.1, 11.6)	**0.024**	0.335

Data are presented as descriptive means with 95% confidence intervals and as unadjusted raw mean differences (MHOO minus MUOO) with 95% confidence intervals. Bold *p* values denote statistically significant differences (*p* < 0.05). Two-sided *p* values are derived from adjusted analyses of covariance (parametric or non-parametric, as appropriate) including sex, age, and body mass index as covariates. Therefore, the confidence intervals for raw mean differences are descriptive and are not expected to correspond directly to the reported adjusted *p* values. ANGPTL3, angiopoietin-like 3; ANGPTL4, angiopoietin-like 4; DPP4, dipeptidyl peptidase-4; GDF15, growth differentiation factor 15; RBP4, retinol-binding protein 4; SHBG, sex hormone-binding globulin.

Moreover, participants with MHOO showed higher sex hormone-binding globulin (SHBG) levels (mean difference: 5.7 nmol/L; 95% CI, −0.1 to 11.6; *p* = 0.024; Cohen’s d = 0.335) than MUOO (Table 3). No significant between-group differences were detected for angiopoietin-like 3 (ANGPTL3), angiopoietin-like 4 (ANGPTL4), β-Klotho, clusterin, follistatin, or growth differentiation factor 15 (GDF15) levels (all *p* values ≥ 0.149; Table 3).

### MHOO and MUOO exhibit similar fecal microbiota composition

Despite the clear differences in fat distribution and circulating adipokine/hepatokine levels, we found no significant differences in fecal microbiota alpha (all *p* values ≥ 0.103; Figures S1A–S1C) or beta diversity (*p* = 0.076; Figure S1D) between MHOO and MUOO. The overall taxonomic composition was similar between groups, predominantly represented by the genera *Bacteroides*, *Faecalibacterium*, *Subdoligranulum*, and *Oscillospiraceae_UCG-002* (Figure S1E).

### VAT and hepatic fat are major distinguishing factors of MUOO from MHOO

To identify the most discriminative features of the MHOO phenotype, we integrated all 39 outcomes using principal component analysis (PCA) (Table S1). PCA revealed partial separation between MHOO and MUOO groups along principal component 1 (PC1; Figure 3A). The variables contributing most to PC1 included (1) anthropometric and body composition variables: waist-to-hip ratio, neck and waist circumferences, mid-thigh skeletal muscle tissue, fat-free mass, and fat mass; (2) specific abdominal adipose tissue depots: VAT volume and the abdominal VAT-to-SAT ratio; (3) mid-thigh SAT area; and (4) hepatic fat fraction (Figure 3A). To further identify the most discriminative features between groups, we applied sparse partial-least-squares discriminant analysis (sPLS-DA), which revealed that VAT volume and hepatic fat fraction were the greatest discriminants between MHOO and MUOO (Figure 3B).

**Figure 3:**
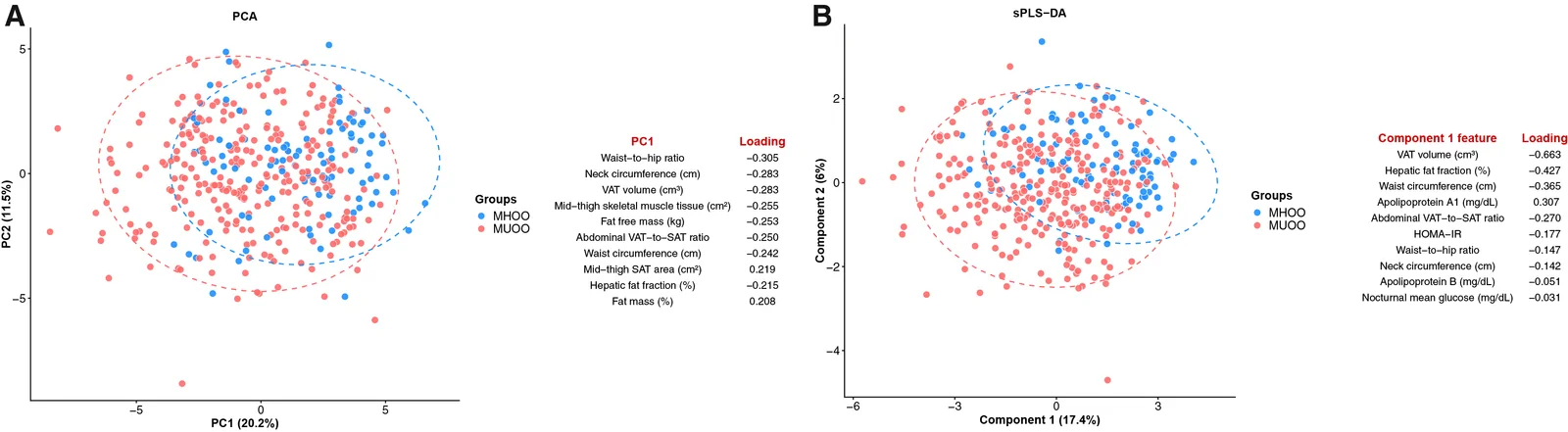
Dimensionality reduction analysis of features differentiating the MHOO and MUOO phenotypes in participants from the combined study I and study II cohorts (A) Principal component analysis (PCA) showing principal component 1 (PC1) and the ten features with the greatest loading coefficients among 39 outcomes assessed across the full sample (Table S1). Variables used to define the phenotypes were excluded from this analysis. (B) Sparse partial-least-squares discriminant analysis (sPLS-DA) of participants with MHOO and MUOO, along with the loading coefficients of the ten features used to construct component 1. HOMA-IR, homeostasis model assessment of insulin resistance; IMAT, intermuscular adipose tissue; PC2, principal component 2; SAT, subcutaneous adipose tissue; VAT, visceral adipose tissue.

Next, we conducted similar analyses in the study I cohort alone, which included a total of 56 outcomes (Table S1), as this was the only subsample with available data on adipokines, hepatokines, and fecal microbiota composition. The variables contributing most to PC1 included (1) anthropometric and body composition variables: waist-to-hip ratio, neck and waist circumferences, mid-thigh skeletal muscle tissue, fat-free mass, and fat mass; (2) specific abdominal adipose tissue depots: VAT volume and abdominal VAT-to-SAT ratio; (3) mid-thigh SAT area; and (4) 24-h mean glucose levels (Figure S2A). sPLS-DA analysis identified apolipoprotein B and neck circumference as top discriminants, followed by VAT volume, between MHOO and MUOO in this subsample (Figure S2B).

## Discussion

In this integrative deep-phenotyping study of adults with overweight/obesity, we show that the MHOO phenotype is characterized by a distinct profile of fat distribution and circulating biomarkers compared with its metabolically unhealthy counterpart. Individuals with MHOO exhibited lower visceral and hepatic fat as well as lower fat accumulation in the pancreas and intermuscular depots. These differences were accompanied by distinct levels of selected adipokines and hepatokines, suggesting a more favorable biological context despite excess adiposity.

Our results reinforce the expandability hypothesis, suggesting that MHOO individuals maintain metabolic health through the preferential expansion of SAT,^11^^,^^27^ particularly in the mid-thigh area,^5^ which may facilitate safer lipid storage and limit ectopic fat deposition in other organs.^4^^,^^5^^,^^16^ While the role of VAT and hepatic fat in metabolic dysfunction is well documented,^3^^,^^4^^,^^21^^,^^28^^,^^29^^,^^30^^,^^31^ our study extends this framework to include pancreatic fat and IMAT. The lower pancreatic fat observed in MHOO is consistent with literature linking pancreatic steatosis to metabolic dysfunction and may indicate a more favorable pancreatic metabolic profile, even before overt dysglycemia develops.^14^^,^^16^ Furthermore, the divergence between abdominal and mid-thigh IMAT suggests localized differences in metabolic vulnerability, and lower IMAT in MHOO may reflect more favorable skeletal muscle insulin sensitivity and lower tissue-specific inflammation.^13^ Notably, VAT volume and hepatic fat emerged as the strongest discriminators between phenotypes. Overall, our findings underscore that fat distribution, rather than total adiposity, is a key feature distinguishing MHOO from MUOO, an aspect still underrecognized in current definitions of metabolic health in obesity.^5^

This pattern of fat distribution was accompanied by distinct levels of selected circulating adipokines and hepatokines. Higher adiponectin and SHBG levels in MHOO are compatible with a more anti-inflammatory and insulin-sensitizing metabolic profile.^17^^,^^32^ Conversely, higher RBP4 levels in MUOO may reflect impaired adipose tissue-liver signaling and a more adverse metabolic state, given its reported associations with inflammation, insulin resistance, and altered hepatic glucose metabolism.^33^ Together, these findings suggest that differences in liver-adipose tissue signaling may be associated with the preservation of metabolic health in MHOO.

Interestingly, despite the clear divergence in fat distribution and circulating biomarkers, we observed no significant differences in fecal microbiota diversity or composition between MHOO and MUOO. This may suggest that, at this stage of obesity, differences in fat partitioning and circulating adipokine/hepatokine levels are more strongly associated with metabolic phenotype than fecal microbiota composition. While other studies in geographically distinct cohorts have reported microbial shifts,^20^^,^^25^ our findings suggest that, in this population, metabolic resilience may be more closely related to host-derived factors than to detectable differences in fecal microbiota composition.

In summary, adults with MHOO exhibit a more favorable fat distribution and a distinct adipokine/hepatokine profile. Visceral and hepatic fat emerged as the strongest discriminative features of this phenotype, supporting their potential value for improving cardiometabolic risk stratification beyond BMI in adults with overweight or obesity.

### Limitations of the study

Our study has limitations, including its cross-sectional design, the lack of a normal-weight reference group, and the dependence of the MHOO concept on the criteria and thresholds applied, which may influence phenotype classification.^34^ Accordingly, the absence of predefined cardiometabolic abnormalities should not necessarily be interpreted as optimal metabolic health across all risk variables. Nevertheless, by integrating MRI with deep phenotyping, we provide a comprehensive characterization of metabolic health in overweight and obesity. These findings highlight the importance of considering not only the amount of fat but also its distribution and relationship with circulating biological markers.

## Resource availability

### Lead contact

Requests for further information and resources should be directed to and will be fulfilled by the lead contact, Antonio Clavero-Jimeno (claveroa@ugr.es).

### Materials availability

This study did not generate new unique reagents.

### Data and code availability

Raw 16S rRNA gene amplicon sequencing data have been deposited in the NCBI Sequence Read Archive (SRA) and are publicly available as of the date of publication. The BioProject accession number PRJNA1287213 is listed in the key resources table. The SILVA 138.1 SSU reference database used for taxonomic classification is also publicly available, with the corresponding resource information and access link provided in the key resources table. All other data reported in this paper will be shared by the lead contact upon request. This paper does not report original code. Any additional information required to reanalyze the data reported in this paper is available from the lead contact upon request.

## Acknowledgments

This study was funded by grant PID2022.141506OB.I00, funded by MCIU/AEI/10.13039/501100011033 and ERDF, EU to J.R.R.; Junta de Andalucía, Consejería de Transformación Económica, Industria, Conocimiento y Universidades (A-CTS-516-UGR20) to J.R.R.; the University of Granada Plan Propio de Investigación-Excellence actions: Unit of Excellence on Exercise Nutrition and Health (UCEENS) to J.R.R.; Instituto de Salud Carlos III, MCIU/AES and ERDF, EU A Way of Making Europe (project reference: PI24/01360), the Consejo Superior de Deportes, Ministerio de Cultura y Deporte (project reference: EXP_75091), and Plan de Recuperación, Transformación y Resiliencia-funded by the European Union – NextGenerationEU to I.L.; the Government of Navarra, Departamento de Desarrollo Economico y Empresarial (0011-1365-2021-00070) and Plan de Promoción de Grupos de Investigación de la Universidad Pública de Navarra to I.L.; and Spanish Ministry of Universities (FPU21/01161 to A.C.-J., FPU22/01631 to J.J.M.-O., FPU23/02158 to M.M.-F., and FPU18/03357 to M.D.-M.). A.C.-C. is supported by the Ministerio de Ciencia, Innovación y Universidades, European Union NextGenerationEU/PRTR (FJC2020-043385-I) and by the Junta de Andalucía, Consejería de Salud y Consumo (RHJ-0098-2024). A.C.-M. is supported by MCIN/AEI/10.13039/501100011033, the European Union “NextGenerationEU”/PRTR (JDC2022-048679-I), and the Horizon Europe Programme under the Marie Skłodowska Curie grant agreement no. 101207146. A.L.-P. received a Sara Borrell Contract from the Spanish Ministry of Health (CD22/00109) and a Researcher Contract from Foundation of the Spanish Association Against Cancer (FAECC) (INVES2610029LOPE). In addition, funding was provided by the EXERNET Research Network on Exercise and Health (DEP2005-00046/ACTI; 09/UPB/19; 45/UPB/20; 27/UPB/21 to I.L. and J.R.R.). This work is part of a doctoral thesis conducted in the official Doctoral Program in Biomedicine at the University of Granada, Spain. The funders had no role in the design, conduct, or interpretation of the study. The graphical abstract was created in BioRender. Ruiz, J. (2026) https://BioRender.com/b80jhji.

## Author contributions

Study concept and design, A.C.-J. and J.R.R.; acquisition of data, A.C.-J., J.J.M.-O., A.G., A.C.-M., A.C.-C., M.D.-M., M.M.-F., and A.L.-V.; analysis and interpretation of data, A.C.-J., A.G., A.C.-M., and J.R.R.; drafting of the manuscript, A.C.-J.; critical revision of the manuscript, A.C.-J., J.J.M.-O., A.G., A.C.-M., A.C.-C., L.J.-F., M.D.-M., M.M.-F., A.L.-V., A.L.-P., M.A.A., E.P., A.Z., I.U., M.C., R.C., J.L.M.-R., I.L., and J.R.R.; statistical analysis, A.C.-J., A.G., and A.C.-M.; funding, I.L. and J.R.R.; administrative, technical, or material support, I.L. and J.R.R.; study supervision, J.R.R.

## Declaration of interests

The authors declare no competing interests.

## Declaration of generative AI and AI-assisted technologies in the writing process

During the preparation of this work, the authors used ChatGPT (OpenAI) to assist with language editing and improve the clarity and readability of the manuscript. After using this tool, the authors reviewed and edited the content as needed and take full responsibility for the content of the publication.

## STAR★Methods

### Key resources table

**Table:** 

REAGENT or RESOURCE	SOURCE	IDENTIFIER
**Biological samples**		

Venous blood samples from Study I and Study II participants	Study participants recruited in Granada and Pamplona, Spain	ClinicalTrials.gov: NCT05310721; NCT05897073
Fecal samples from Study I participants	Study participants recruited in Granada and Pamplona, Spain	ClinicalTrials.gov: NCT05310721

**Critical commercial assays**		

Human adiponectin DuoSet ELISA	R&D Systems	Cat# DY1065
Human DPP4/CD26 DuoSet ELISA	R&D Systems	Cat# DY1180
Human IL-6 Quantikine HS ELISA	R&D Systems	Cat# HS600C
Human leptin DuoSet ELISA	R&D Systems	Cat# DY398
Human RBP4 DuoSet ELISA	R&D Systems	Cat# DY3378
Human ANGPTL3 DuoSet ELISA	R&D Systems	Cat# DY3829
Human clusterin DuoSet ELISA	R&D Systems	Cat# DY5874
Human SHBG DuoSet ELISA	R&D Systems	Cat# DY2656
Human omentin DuoSet ELISA	R&D Systems	Cat# DY4254
Human resistin DuoSet ELISA	R&D Systems	Cat# DY1359
Human ANGPTL4 DuoSet ELISA	R&D Systems	Cat# DY3485
Human β-Klotho DuoSet ELISA	R&D Systems	Cat# DY5889
Human follistatin DuoSet ELISA	R&D Systems	Cat# DY669
Human GDF15 DuoSet ELISA	R&D Systems	Cat# DY957
QIAamp PowerFecal Pro DNA Kit	Qiagen	Cat# 51804

**Deposited data**		

Raw 16S rRNA gene amplicon sequencing data	NCBI Sequence Read Archive (SRA)	BioProject: PRJNA1287213
SILVA 138.1 SSU reference database	SILVA ribosomal RNA database project	Release 138.1: https://www.arb-silva.de/documentation/release-1381/

**Oligonucleotides**		

16S rRNA V3–V4 forward primer 341F	Illumina	5′TCGTCGGCAGCGTCAGATGT GTATAAGAGACAGCCTACGGG NGGCWGCAG
16S rRNA V3–V4 reverse primer 805R	Illumina	5′GTCTCGTGGGCTCGGAGATGTG TATAAGAGACAGGACTACHVGGG TATCTAATCC

**Software and algorithms**		

R	R Foundation for Statistical Computing	v4.5.3
QIIME 2	Caporaso Lab	v2024.10
DADA2	Caporaso Lab	QIIME 2 plugin
ANCOM-BC2	ANCOMBC Bioconductor package	v2.8.1
ggplot2	CRAN	v3.5.2
Durga	CRAN	v2.0
emmeans	CRAN	v1.10.1
vegan	CRAN	v2.6-10
adonis2	vegan R package	v2.6-10
missMDA	CRAN	v1.2.0
mixOmics	Bioconductor	v6.32.0
3D Slicer software (v. 5.8.1)	3D slicer (Kitware)	https://www.slicer.org/
TisSeg	In-house software	N/A
ActiLife	ActiGraph LLC	v6.13.6
GGIR	CRAN	v3.1-8
GGIRmatcher	PROFITH research group	https://github.com/PROFITH/GGIRmatcher
EasyDiet	Biocentury, S.L.U.	2016
EvalFINUT	Fundación Iberoamericana de Nutrición (FINUT)	https://www.finut.org/evalfinut/

**Other**		

Electronic column scale and stadiometer	SECA	Model 799
QDR-4500W dual-energy X-ray absorptiometer	Hologic Inc.	QDR-4500W
Horizon Wi dual-energy X-ray absorptiometer	Hologic Inc.	Horizon Wi
Multi-frequency bioelectrical impedance analyzer	Tanita	MC-980MA Plus
Automatic blood pressure monitor	Omron Healthcare Europe B.V.	M3 Comfort
AU5800 clinical chemistry analyzer	Beckman Coulter	AU5800
UniCel DxI 800 immunoassay system	Beckman Coulter	UniCel DxI 800
HA-8180V glycated hemoglobin analyzer	A. Menarini Diagnostics	HA-8180V
Alinity c clinical chemistry system	Abbott Laboratories	Alinity c
G11 automated glycohemoglobin analyzer	Horiba	G11
FreeStyle Libre Pro continuous glucose monitoring system	Abbott Laboratories	FreeStyle Libre Pro
FreeStyle Libre 2 continuous glucose monitoring system	Abbott Laboratories	FreeStyle Libre 2
Triaxial accelerometer	ActiGraph LLC	GT3X+
Magnetom Vida 3-T MRI scanner	Siemens Healthineers	Magnetom Vida
NextSeq 1000 sequencing system	Illumina	NextSeq 1000

### Experimental model and study participant details

#### Study design

The present cross-sectional study used data from the baseline assessments of two randomized controlled trials (RCTs) that followed harmonized core assessment procedures: Study I (ClinicalTrials.gov identifier: NCT05310721) and Study II (NCT05897073). Study I was a multicenter RCT conducted in Granada (southern Spain) and Pamplona (northern Spain),^35^ while Study II was a single-center RCT conducted in Granada.^36^ Detailed descriptions of the trial rationale, design, and protocols are available elsewhere.^36^^,^^37^ Both studies were approved by the appropriate regulatory authorities and ethics committees, and all participants provided written informed consent.^36^^,^^37^ The study protocols followed the most recent revision of the Declaration of Helsinki, and the present study was reported in accordance with the STROBE guidelines.^38^

#### Participants

For Study I, participants were recruited between April 11, 2022, and December 5, 2022, with a study completion date of March 6, 2023. For Study II, recruitment took place between May 2, 2023, and April 6, 2024, with a study completion date of September 5, 2025. Participants were enrolled through advertisements in newspapers and social media, the Endocrinology and Nutrition departments of the *Hospital Universitario Clínico San Cecilio* and *Hospital Virgen de las Nieves* in Granada, the University of Granada community, and the *Hospital Universitario de Navarra* in Pamplona. The present analysis included 384 participants (193 men and 191 women) aged 25–65 years with overweight/obesity, defined as a body mass index (BMI) ≥25.0 and <40.0 kg/m^2^, who did not meet the World Health Organization physical activity recommendations (i.e., engaging in <150 min/week of MVPA).^39^ Exclusion criteria were: i) history of major adverse cardiovascular events or diagnosis of diabetes or cardiovascular disease; ii) major sleep or eating disorders; iii) shift work involving night shifts; iv) pregnancy or lactation; v) use of medications affecting glucose metabolism; and vi) claustrophobia preventing MRI assessment.

#### Metabolic phenotype classification

Participants were retrospectively classified as MHOO or MUOO using previously proposed criteria that have been widely applied in the literature.^40^ MUOO was defined as having BMI ≥25 kg/m^2^ together with at least one of the following comorbidities: a) fasting triglyceride levels ≥150 mg/dL or use of lipid-lowering medication; b) fasting high-density lipoprotein cholesterol (HDL-C) levels <40 mg/dL in men and <50 mg/dL in women, or use of lipid-lowering medication; c) resting systolic blood pressure ≥130 mmHg or diastolic blood pressure ≥85 mmHg, or use of antihypertensive medication; d) fasting glucose levels ≥100 mg/dL or use of glucose-lowering medication. MHOO was defined as BMI ≥25 kg/m^2^ without any of these risk factors. Based on these criteria, 99 participants were classified as MHOO and 285 as MUOO.

### Method details

#### MRI-derived specific fat depots

Specific fat depots were assessed under fasting conditions (≥4 h) using a 3-Tesla MRI scanner (Magnetom Vida, Siemens Healthineers, Erlangen, Germany). VAT, IMAT, and SAT volumes were quantified from Dixon MR images in the abdominal region between the L3 and L5 vertebrae, whereas mid-thigh IMAT, SAT, and skeletal muscle areas were quantified from an axial image acquired at the mid-femur level.^15^ Fat and water image series were processed using a semi-automatic in-house library called TisSeg, developed within our research group, together with its plugin for the open-source software 3D Slicer (www.slicer.org). This library enabled automatic segmentation of the selected slices. The abdominal VAT-to-SAT ratio was also calculated. Hepatic fat fraction was determined through manual slice-by-slice segmentation of the whole liver, excluding major vessels and surrounding non-hepatic tissues, followed by volumetric quantification of the mean fat fraction.^41^ Pancreatic fat fraction was assessed by placing regions of interest in the head, body, and tail of the pancreas, avoiding visible vessels and surrounding adipose tissue. The median value was obtained for each region, and the overall pancreatic fat fraction was calculated as the mean of the three regional values.

#### Adipokines and hepatokines

Circulating peripheral blood adipokine and hepatokine concentrations were quantified exclusively in the Study I cohort using commercial Enzyme-Linked Immunosorbent Assay (ELISA) kits (R&D Systems, MN, USA), following the manufacturer’s instructions. Serum was used for measurements of: adiponectin (DY1065), DPP4 (DY1180), IL-6 (HS600C), leptin (DY398), RBP4 (DY3378), ANGPTL3 (DY3829), clusterin (DY5874), and SHBG (DY2656). Plasma was used for measurements of: omentin (DY4254), resistin (DY1359), ANGPTL4 (DY3485), β-Klotho (DY5889), follistatin (DY669), and GDF15 (DY957). Based on the current literature on adipokine and hepatokine classification,^17^^,^^18^^,^^42^ and considering their predominant tissue source and metabolic functions, adiponectin, DPP4, IL-6, leptin, omentin, resistin, and RBP4 were categorized as adipokines, whereas ANGPTL3, ANGPTL4, β-Klotho, clusterin, follistatin, GDF15, and SHBG were categorized as hepatokines.

#### Fecal microbiota

Fecal microbiota analysis was exclusively performed on 182 participants from the Study I cohort. Participants self-collected fecal samples in sterile containers and delivered them to the research center, where samples were stored at −80°C until analysis. Bacterial DNA was isolated following the protocol of the QIAamp PowerFecal Pro DNA Kit (Qiagen, Hilden, Germany). The V3-V4 hypervariable region of the 16S rRNA gene (341F and 805R primers) was sequenced using a read length of 2 x 300 bp paired-end run on a NextSeq1000-Illumina platform at the Microbiology Sequencing Service of the *Hospital Universitario Clínico San Cecilio* (Granada, Spain), following Illumina protocols (Illumina Inc., San Diego, CA, USA). Post-sequencing analyses were conducted using the Quantitative Insights Into Microbial Ecology (QIIME2) (v.2024.10) pipeline,^43^ applying the DADA2 algorithm^44^ to infer the Amplicon Sequence Variants (ASVs). Taxonomic classification was assigned using a pre-trained classifier based on the SILVA 138.1 SSU reference database,^45^ considering the genus-level taxonomy as the highest classification category. Ten samples were removed due to low sequencing quality, resulting in a final dataset of 172 samples with a mean ± standard deviation (SD) of 303,404 ± 164,728 reads per sample (MHOO [*n* = 45; 17 men and 28 women] and MUOO [*n* = 127; 70 men and 57 women]). ASVs present in fewer than 10% of samples were removed. Alpha diversity (observed features, Shannon entropy, and Simpson index) and beta diversity (Bray-Curtis dissimilarity) metrics were estimated based on the random selection of 38,500 reads per sample.

#### Anthropometry and body composition

Before body composition and cardiometabolic assessments, participants were (i) instructed to fast for approximately 10 h, consuming only water, (ii) abstain from alcohol and diuretics for 24 h, (iii) avoid caffeine and other stimulants for 12 h, (iv) avoid moderate-intensity physical activity for 24 h, and (v) avoid vigorous-intensity physical activity for 48 h.

Body weight and height were measured in the morning with participants barefoot and wearing light clothing using a SECA 799 electronic column scale and stadiometer (SECA, Hamburg, Germany). BMI was calculated as body weight in kilograms divided by height in meters squared. Neck, waist, and hip circumferences were measured by trained personnel following International Society for the Advancement of Kinanthropometry procedures.^46^

Fat-free mass (kg) and fat mass (kg and %) were assessed in the morning after an overnight fast. In Study I, body composition was measured by dual-energy X-ray absorptiometry using a Hologic QDR-4500W scanner in Granada and a Hologic Horizon Wi scanner in Pamplona (Hologic Inc., Bedford, MA, USA). In Study II, body composition was assessed using multi-frequency bioelectrical impedance analysis with a Tanita MC-980MA Plus analyzer (Tanita, Tokyo, Japan).

#### Blood pressure, glucose homeostasis, blood lipid profile, liver enzymes, and physical activity

Resting systolic and diastolic blood pressure were measured in the morning using an automatic M3 Comfort monitor (Omron Healthcare Europe B.V., Hoofddorp, The Netherlands), following the 2021 European Society of Hypertension recommendations.^47^ Three consecutive measurements were obtained after a brief rest period, with a 1-min interval between measurements, and the mean of the last two readings was used for analysis.

Venous blood samples were collected in the morning after an overnight fast, processed according to standardized clinical laboratory procedures, and stored at −80°C until analysis. Glucose, insulin, HbA1c, total cholesterol, HDL-C, triglycerides, apolipoprotein A1, apolipoprotein B, ALT, GGT, and alkaline phosphatase were measured using automated analyzers. In Study I, biochemical analyses were performed using AU5800 and UniCel DxI 800 systems (Beckman Coulter, Brea, CA, USA), and HbA1c was measured using an HA-8180V analyzer (A. Menarini Diagnostics, Florence, Italy).^37^ In Study II, analyses were performed using an Alinity c system (Abbott Laboratories, Abbott Park, IL, USA), an UniCel DxI 800 immunoassay system, and a G11 glycohemoglobin analyzer (Horiba, Kyoto, Japan).^36^ LDL-C was estimated as total cholesterol − HDL-C − triglycerides/5, and HOMA-IR was calculated as fasting insulin × fasting glucose/405.

Participants’ daily glucose levels and physical activity were simultaneously monitored for 14 consecutive days. Interstitial glucose was measured using continuous glucose monitoring (CGM) devices (FreeStyle Libre Pro in Granada and FreeStyle Libre 2 in Pamplona; Abbott Laboratories, Chicago, IL, USA), which stored average glucose values approximately every 15 min^48^ During the same period, participants wore a triaxial accelerometer (ActiGraph GT3X+, ActiGraph LLC, Pensacola, FL, USA) on the non-dominant wrist 24 h/day, initialized to record raw accelerations at 100 Hz. Accelerometer data were downloaded using ActiLife software (v6.13.6; ActiGraph LLC) and processed using the open-source GGIR R package (v3.1-8). Briefly, the Euclidean Norm Minus One was calculated over 5-s epochs, with negative values rounded to zero. Non-wear periods were identified from the magnitude and variability of raw acceleration across the three axes and imputed, when appropriate, using the average acceleration recorded at the same time on the remaining days. Sleep and waking periods were identified using an automated algorithm based on arm-posture variability and guided by participant-reported sleep times. MVPA was defined as waking-time acceleration ≥100 m*g* accumulated in bouts of at least 5 min, allowing up to 20% of each bout below this threshold. CGM and accelerometer data were time-matched using GGIRmatcher (https://github.com/PROFITH/GGIRmatcher). Days were considered valid when CGM data were available for at least 70% of the day and accelerometer wear time was ≥16 h. Accelerometer-derived sleep onset and wake-up times were used to calculate participant-level mean glucose over the full day (24-h mean glucose), waking hours (diurnal mean glucose), and sleeping hours (nocturnal mean glucose), together with their corresponding CVs.^49^ Sedentary time, LPA, and MVPA were also derived from accelerometer data and averaged across valid monitoring days. As participants reported not engaging in structured exercise, measured MVPA was considered to reflect lifestyle physical activity.

#### Dietary intake

Energy (kcal/day) and macronutrient intake (% of total energy intake) were assessed using three nonconsecutive 24-h dietary recalls, including two working days and one non-working day. Recalls were administered through face-to-face or online interviews conducted by trained research nutritionists. In Study I, dietary intake was estimated using EasyDiet®—Programa de Gestión de la Consulta de la Academia Española de Nutrición y Dietética (Biocentury, S.L.U., Barcelona, Spain).^37^ In Study II, dietary intake was estimated using EvalFINUT® (https://www.finut.org/evalfinut/), based on the Spanish Food Composition Database (BEDCA).^36^

Mediterranean dietary pattern adherence was assessed using the validated 14-item MEDAS questionnaire, a food-based indicator of overall diet quality. Total scores range from 0 to 14, with higher scores indicating greater adherence to the Mediterranean dietary pattern.^50^

### Quantification and statistical analysis

#### Statistical analysis

The sample size of the original trials was determined based on their prespecified primary outcomes.^36^^,^^37^ As this study is a secondary analysis of those trials, no additional sample size calculation was performed.

Continuous variables are presented as mean (SD), while categorical variables are presented as number (percentage) of participants. Group comparisons between participants with MHOO and MUOO were conducted using analysis of covariance (ANCOVA) for continuous outcomes and chi-square tests for categorical variables. ANCOVA models were adjusted for age, sex, and BMI, except for MRI-derived specific fat-depot outcomes, which were adjusted for age, sex, and whole-body fat mass. Depending on the distribution of the data (assessed through Shapiro-Wilk tests and distribution plots), either parametric ANCOVA or non-parametric rank-based ANCOVA was applied.^51^ Additional exploratory analyses stratified by sex (i.e., separately in men and women) were conducted for specific fat depots using the same ANCOVA models, adjusted for age and whole-body fat mass. To account for multiple testing, *p* values for the MHOO versus MUOO group effect across all outcomes were adjusted using the Holm procedure.

Differences in fecal microbiota beta diversity among groups were evaluated by permutational multivariate analysis of variance (PERMANOVA) with 999 permutations. Principal coordinates analysis (PCoA) was applied to visualize sample clustering based on Bray-Curtis dissimilarity. Differential abundance analysis at the genus level was performed using the Analysis of Compositions of Microbiomes with Bias Correction 2 (ANCOM-BC2) framework.^52^ A sensitivity analysis was conducted to assess the impact of pseudo-counts addition on zero counts, reducing the likelihood of false positives. Sex, age and BMI were used as covariates. Taxa with an adjusted *q*-value <0.05 after Holm-Bonferroni correction and that passed the sensitivity analysis were considered significantly differentially abundant.

To identify multivariate patterns distinguishing the MHOO and MUOO phenotypes, we used two complementary dimensionality reduction approaches: (i) PCA, to explore the overall data structure in an unsupervised manner, and (ii) sPLS-DA, to identify the variables that best discriminated between phenotypes. First, PCA was applied across the full sample (Studies I and II combined), excluding variables used to define phenotype classification (i.e., body weight, height, BMI, triglycerides, HDL-C, blood pressure, fasting glucose, and medication usage), resulting in a total of 39 outcomes (i.e., including body circumferences, body composition, glucose homeostasis, blood lipid profile, liver enzymes, sedentary time and physical activity, dietary intake, and specific fat depots) (Table S2). All variables were auto-scaled (mean-centered and unit-variance scaled) before analysis. Missing values were imputed using an iterative PCA approach based on a regularized expectation maximization algorithm.^53^ Next, sPLS-DA was conducted with MHOO and MUOO data using 2 components and 10 variables per component, an algorithm designed to identify a small subset of features that best discriminate between groups.^54^ Complementary analyses were then performed in the Study I cohort alone. In this subsample, PCA and sPLS-DA included all variables used in the pooled analysis, along with additional adipokines, hepatokines, and alpha diversity indices derived from fecal microbiota (*n* = 56 outcomes) (Table S2).

All tests were two-sided, with statistical significance set at *p* < 0.05. Analyses were conducted using R software, v4.5.3 (R Foundation for Statistical Computing, Vienna, Austria; https://cran.r-project.org/). Plots were generated using *ggplot2 (v3.5.2)* and *Durga (v2.0) packages* in R, and regression diagnostics were performed with the *emmeans package (v1.10.1)*. PERMANOVA was performed using the *vegan package (v2.6.10)* and the adonis2 function using default settings (999 permutations). ANCOM-BC2 was carried out using the *ANCOMBC package (v2.8.1)*. PCA was conducted using the *stats package (v4.5.3),* and missing data were imputed using the *missMDA package (v1.2.0)*. sPLS-DA analysis was performed using the *MixOmics package (v6.32.0)*.

## References

[1] GBD 2021 Adult BMI Collaborators (2025). Global, regional, and national prevalence of adult overweight and obesity, 1990-2021, with forecasts to 2050: a forecasting study for the Global Burden of Disease Study 2021. Lancet.

[2] Valenzuela P.L., Carrera-Bastos P., Castillo-García A., Lieberman D.E., Santos-Lozano A., Lucia A. (2023). Obesity and the risk of cardiometabolic diseases. Nat. Rev. Cardiol..

[3] Tanriover C., Copur S., Gaipov A., Ozlusen B., Akcan R.E., Kuwabara M., Hornum M., Van Raalte D.H., Kanbay M. (2023). Metabolically healthy obesity: Misleading phrase or healthy phenotype?. Eur. J. Intern. Med..

[4] Stefan N., Häring H.-U., Hu F.B., Schulze M.B. (2013). Metabolically healthy obesity: epidemiology, mechanisms, and clinical implications. Lancet Diabetes Endocrinol..

[5] Schulze M.B., Stefan N. (2024). Metabolically healthy obesity: from epidemiology and mechanisms to clinical implications. Nat. Rev. Endocrinol..

[6] Blüher M. (2020). Metabolically Healthy Obesity. Endocr. Rev..

[7] Zhou Z., Macpherson J., Gray S.R., Gill J.M.R., Welsh P., Celis-Morales C., Sattar N., Pell J.P., Ho F.K. (2021). Are people with metabolically healthy obesity really healthy? A prospective cohort study of 381,363 UK Biobank participants. Diabetologia.

[8] Mongraw-Chaffin M., Foster M.C., Anderson C.A.M., Burke G.L., Haq N., Kalyani R.R., Ouyang P., Sibley C.T., Tracy R., Woodward M., Vaidya D. (2018). Metabolically Healthy Obesity, Transition to Metabolic Syndrome, and Cardiovascular Risk. J. Am. Coll. Cardiol..

[9] Rubino F., Cummings D.E., Eckel R.H., Cohen R.V., Wilding J.P.H., Brown W.A., Stanford F.C., Batterham R.L., Farooqi I.S., Farpour-Lambert N.J. (2025). Definition and diagnostic criteria of clinical obesity. Lancet Diabetes Endocrinol..

[10] Jurado-Fasoli L., Sanchez-Delgado G., Alcantara J.M.A., Acosta F.M., Sanchez-Sanchez R., Labayen I., Ortega F.B., Martinez-Tellez B., Ruiz J.R. (2024). Adults with metabolically healthy overweight or obesity present more brown adipose tissue and higher thermogenesis than their metabolically unhealthy counterparts. EBioMedicine.

[11] Neeland I.J., Ross R., Després J.-P., Matsuzawa Y., Yamashita S., Shai I., Seidell J., Magni P., Santos R.D., Arsenault B. (2019). Visceral and ectopic fat, atherosclerosis, and cardiometabolic disease: a position statement. Lancet Diabetes Endocrinol..

[12] Neeland I.J., Lim S., Tchernof A., Gastaldelli A., Rangaswami J., Ndumele C.E., Powell-Wiley T.M., Després J.-P. (2024). Metabolic syndrome. Nat. Rev. Dis. Primers.

[13] Goodpaster B.H., Bergman B.C., Brennan A.M., Sparks L.M. (2023). Intermuscular adipose tissue in metabolic disease. Nat. Rev. Endocrinol..

[14] Wagner R., Eckstein S.S., Yamazaki H., Gerst F., Machann J., Jaghutriz B.A., Schürmann A., Solimena M., Singer S., Königsrainer A. (2022). Metabolic implications of pancreatic fat accumulation. Nat. Rev. Endocrinol..

[15] Camacho-Cardenosa A., Clavero-Jimeno A., Gatti A., Dote-Montero M., Concepción M., Alfaro-Magallanes V.M., Martin-Olmedo J.J., Cabeza R., Idoate F., Martín-Rodríguez J.L. (2025). Impact of Abdominal and Thigh Intermuscular Adipose Tissue on Glucose and Cardiometabolic Risk in Adults With Obesity. J. Clin. Endocrinol. Metab..

[16] Petrov M.S., Taylor R. (2022). Intra-pancreatic fat deposition: bringing hidden fat to the fore. Nat. Rev. Gastroenterol. Hepatol..

[17] Stefan N., Schick F., Birkenfeld A.L., Häring H.-U., White M.F. (2023). The role of hepatokines in NAFLD. Cell Metab..

[18] Tilg H., Ianiro G., Gasbarrini A., Adolph T.E. (2025). Adipokines: masterminds of metabolic inflammation. Nat. Rev. Immunol..

[19] Carmody R.N., Bisanz J.E. (2023). Roles of the gut microbiome in weight management. Nat. Rev. Microbiol..

[20] Lacruz-Pleguezuelos B., Pérez-Cuervo A., Coleto-Checa D., Bazán G.X., Romero-Tapiador S., Freixer G., Fernández-Cabezas J., Aguilar-Aguilar E., Martín-Segura A., Cárdenas-Roig N. (2026). Network topology of the gut microbiome associates with metabolic health in obesity. Nat. Commun..

[21] Petersen M.C., Smith G.I., Palacios H.H., Farabi S.S., Yoshino M., Yoshino J., Cho K., Davila-Roman V.G., Shankaran M., Barve R.A. (2024). Cardiometabolic characteristics of people with metabolically healthy and unhealthy obesity. Cell Metab..

[22] Reinisch I., Ghosh A., Noé F., Sun W., Dong H., Leary P., Dietrich A., Hoffmann A., Blüher M., Wolfrum C. (2025). Unveiling adipose populations linked to metabolic health in obesity. Cell Metab..

[23] Alizadeh S., Mirzaei K., Mohammadi C., Keshavarz S.A., Maghbooli Z. (2017). Circulating omentin-1 might be associated with metabolic health status in different phenotypes of body size. Arch. Endocrinol. Metab..

[24] Ahl S., Guenther M., Zhao S., James R., Marks J., Szabo A., Kidambi S. (2015). Adiponectin Levels Differentiate Metabolically Healthy vs Unhealthy Among Obese and Nonobese White Individuals. J. Clin. Endocrinol. Metab..

[25] Kim M.-H., Yun K.E., Kim J., Park E., Chang Y., Ryu S., Kim H.-L., Kim H.-N. (2020). Gut microbiota and metabolic health among overweight and obese individuals. Sci. Rep..

[26] Smith G.I., Mittendorfer B., Klein S. (2019). Metabolically healthy obesity: facts and fantasies. J. Clin. Investig..

[27] Iacobini C., Pugliese G., Blasetti Fantauzzi C., Federici M., Menini S. (2019). Metabolically healthy versus metabolically unhealthy obesity. Metabolism.

[28] Stefan N., Kantartzis K., Machann J., Schick F., Thamer C., Rittig K., Balletshofer B., Machicao F., Fritsche A., Häring H.U. (2008). Identification and Characterization of Metabolically Benign Obesity in Humans. Arch. Intern. Med..

[29] Zhang H., Ma Z., Pan L., Xu Y., Shao J., Huang Z., Chen Z., Sun Q., Liu C., Lin M. (2016). Hepatic fat content is a determinant of metabolic phenotypes and increased carotid intima-media thickness in obese adults. Sci. Rep..

[30] Tchernof A., Després J.-P. (2013). Pathophysiology of human visceral obesity: an update. Physiol. Rev..

[31] Blüher M. (2014). Are metabolically healthy obese individuals really healthy?. Eur. J. Endocrinol..

[32] Straub L.G., Scherer P.E. (2019). Metabolic Messengers: Adiponectin. Nat. Metab..

[33] Flores-Cortez Y.A., Barragán-Bonilla M.I., Mendoza-Bello J.M., González-Calixto C., Flores-Alfaro E., Espinoza-Rojo M. (2022). Interplay of retinol binding protein 4 with obesity and associated chronic alterations. Mol. Med. Rep..

[34] Després J.-P. (2022). Taking a closer look at metabolically healthy obesity. Nat. Rev. Endocrinol..

[35] Dote-Montero M., Clavero-Jimeno A., Merchán-Ramírez E., Oses M., Echarte J., Camacho-Cardenosa A., Concepción M., Amaro-Gahete F.J., Alcántara J.M.A., López-Vázquez A. (2025). Effects of early, late and self-selected time-restricted eating on visceral adipose tissue and cardiometabolic health in participants with overweight or obesity: a randomized controlled trial. Nat. Med..

[36] Camacho-Cardenosa A., Clavero-Jimeno A., Martin-Olmedo J.J., Amaro-Gahete F., Cupeiro R., Cejudo M.T.G., García Pérez P.V., Hernández-Martínez C., Sevilla-Lorente R., De-la-O A. (2024). Time-restricted eating and supervised exercise for improving hepatic steatosis and cardiometabolic health in adults with obesity: protocol for the TEMPUS randomised controlled trial. BMJ Open.

[37] Dote-Montero M., Merchan-Ramirez E., Oses M., Echarte J., Clavero-Jimeno A., Alcantara J., Camacho-Cardenosa A., Cupeiro R., Rodríguez-Miranda M.d.L.N., López-Vázquez A. (2024). Efficacy of different 8 h time-restricted eating schedules on visceral adipose tissue and cardiometabolic health: A study protocol. Nutr. Metab. Cardiovasc. Dis..

[38] von Elm E., Altman D.G., Egger M., Pocock S.J., Gøtzsche P.C., Vandenbroucke J.P., STROBE Initiative (2007). The Strengthening the Reporting of Observational Studies in Epidemiology (STROBE) statement: guidelines for reporting observational studies. Lancet.

[39] Bull F.C., Al-Ansari S.S., Biddle S., Borodulin K., Buman M.P., Cardon G., Carty C., Chaput J.-P., Chastin S., Chou R. (2020). World Health Organization 2020 guidelines on physical activity and sedentary behaviour. Br. J. Sports Med..

[40] Ortega F.B., Lavie C.J., Blair S.N. (2016). Obesity and Cardiovascular Disease. Circ. Res..

[41] Camacho-Cardenosa A., Clavero-Jimeno A., López-Vázquez A., Concepción M., Martín-Rodríguez J.L., Labayen I., Ruiz J.R., Cabeza R. (2026). Protocol for volumetric quantification of intrahepatic triglyceride content in adults with obesity. STAR Protoc..

[42] Kucukoglu O., Sowa J.P., Mazzolini G.D., Syn W.K., Canbay A. (2021). Hepatokines and adipokines in NASH-related hepatocellular carcinoma. J. Hepatol..

[43] Bolyen E., Rideout J.R., Dillon M.R., Bokulich N.A., Abnet C.C., Al-Ghalith G.A., Alexander H., Alm E.J., Arumugam M., Asnicar F. (2019). Reproducible, interactive, scalable and extensible microbiome data science using QIIME 2. Nat. Biotechnol..

[44] Callahan B.J., McMurdie P.J., Rosen M.J., Han A.W., Johnson A.J.A., Holmes S.P. (2016). DADA2: High-resolution sample inference from Illumina amplicon data. Nat. Methods.

[45] Glöckner F.O., Yilmaz P., Quast C., Gerken J., Beccati A., Ciuprina A., Bruns G., Yarza P., Peplies J., Westram R., Ludwig W. (2017). 25 years of serving the community with ribosomal RNA gene reference databases and tools. J. Biotechnol..

[46] Silva V.S.d., Vieira M.F.S. (2020). International Society for the Advancement of Kinanthropometry (ISAK) Global: international accreditation scheme of the competent anthropometrist. Rev. bras. cineantropom. desempenho hum..

[47] Stergiou G.S., Palatini P., Parati G., O’Brien E., Januszewicz A., Lurbe E., Persu A., Mancia G., Kreutz R., European Society of Hypertension Council and the European Society of Hypertension Working Group on Blood Pressure Monitoring and Cardiovascular Variability (2021). 2021 European Society of Hypertension practice guidelines for office and out-of-office blood pressure measurement. J. Hypertens..

[48] Battelino T., Alexander C.M., Amiel S.A., Arreaza-Rubin G., Beck R.W., Bergenstal R.M., Buckingham B.A., Carroll J., Ceriello A., Chow E. (2023). Continuous glucose monitoring and metrics for clinical trials: an international consensus statement. Lancet Diabetes Endocrinol..

[49] Clavero-Jimeno A., Dote-Montero M., Migueles J.H., Camacho-Cardenosa A., Oses M., Medina J.E., Alcantara J.M.A., Muñoz-Torres M., Labayen I., Ruiz J.R. (2024). Impact of lifestyle moderate-to-vigorous physical activity timing on glycemic control in sedentary adults with overweight/obesity and metabolic impairments. Obesity.

[50] Martínez-González M.A., García-Arellano A., Toledo E., Salas-Salvadó J., Buil-Cosiales P., Corella D., Covas M.I., Schröder H., Arós F., Gómez-Gracia E. (2012). A 14-item Mediterranean diet assessment tool and obesity indexes among high-risk subjects: the PREDIMED trial. PLoS One.

[51] Conover W.J., Iman R.L. (1982). Analysis of covariance using the rank transformation. Biometrics.

[52] Lin H., Peddada S.D. (2024). Multigroup analysis of compositions of microbiomes with covariate adjustments and repeated measures. Nat. Methods.

[53] Greenacre M., Groenen P.J.F., Hastie T., D’Enza A.I., Markos A., Tuzhilina E. (2022). Principal component analysis. Nat. Rev. Methods Primers.

[54] Lê Cao K.-A., Boitard S., Besse P. (2011). Sparse PLS discriminant analysis: biologically relevant feature selection and graphical displays for multiclass problems. BMC Bioinf..

