# Supplementary material for: Divergent ectopic fat distribution and adipokine/hepatokine levels characterize the metabolically healthy overweight/obesity phenotype
Source: iScience. 2026 Sep 30;29(10):117672. doi: 10.1016/j.isci.2026.117672 (PMC13657101; doi:10.1016/j.isci.2026.117672)
Supplement: Supplementary file 1 [file mmc1.pdf]

iScience, Volume *29*

Supplemental information

Divergent ectopic fat distribution and adipokine/hepatokine levels characterize the metabolically healthy overweight/obesity phenotype

Antonio Clavero-Jimeno, Juan J. Martin-Olmedo, Alessandro Gatti, Adrián Cortés-Martín, Alba Camacho-Cardenosa, Lucas Jurado-Fasoli, Manuel Dote-Montero, Marcos Molina-Fernandez, Alejandro López-Vázquez, Amaya Lopez-Pascual, Matías A. Ávila, Estrella Petrina, Ana Zugasti, Iñigo Urdiain, Mara Concepción, Rafael Cabeza, José L. Martín-Rodríguez, Idoia Labayen, and Jonatan R. Ruiz

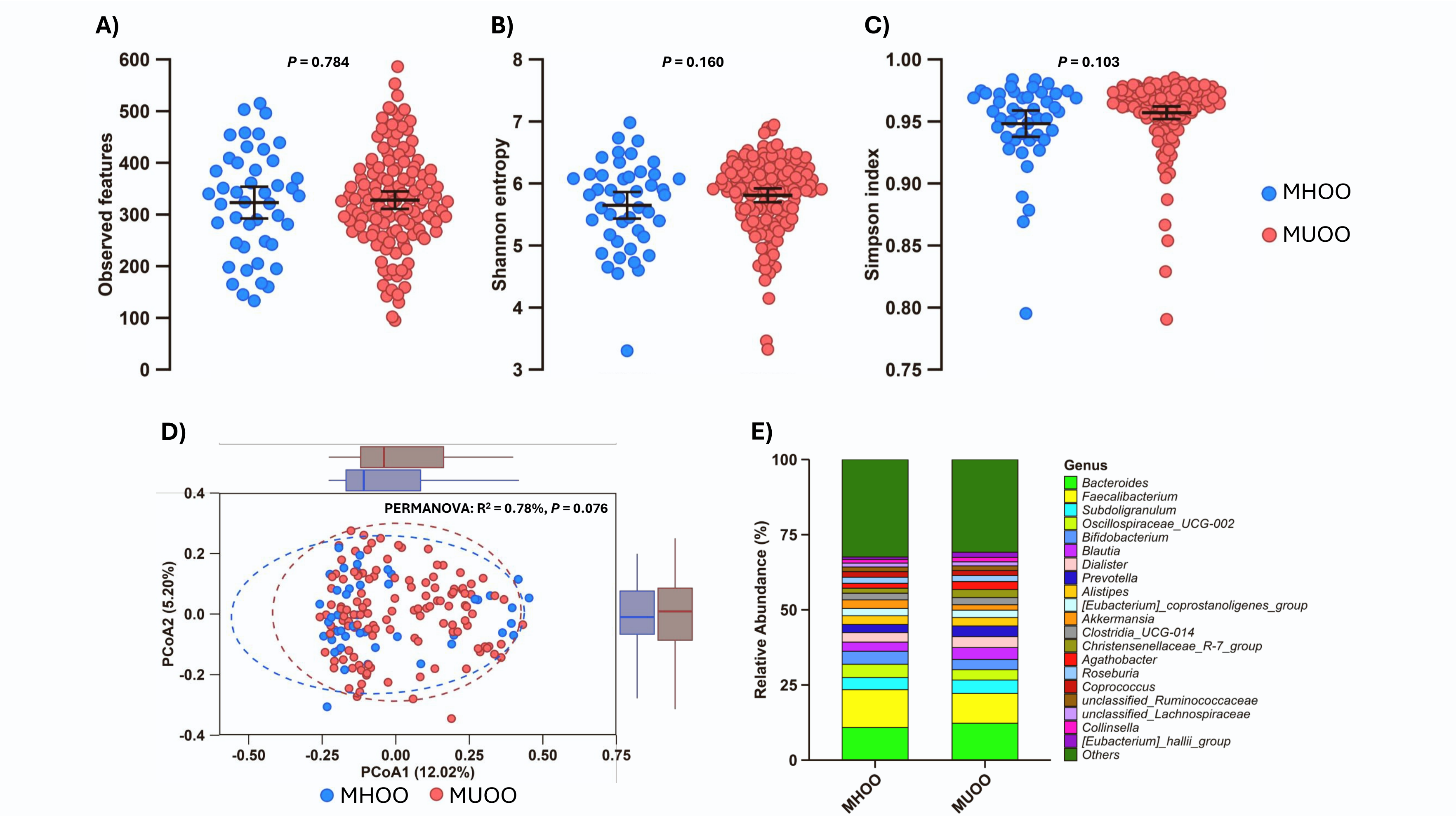

Figure S1. Comparison of fecal microbiota diversity and composition between participants with metabolically healthy overweight/obesity (MHOO; n = 45) and metabolically unhealthy overweight/obesity (MUOO; n = 127). (A-D) Comparisons of alpha and beta diversity indices between MHOO and MUOO participants: **(A)** observed features, **(B)** Shannon entropy, **(C)** Simpson index, and **(D)** Bray-Curtis dissimilarity (*P* = 0.10). No statistically significant differences were detected in alpha or beta diversity between groups. Alpha diversity comparisons were performed using ANCOVA adjusted for age, sex, and body mass index, while beta diversity differences were assessed using PERMANOVA. **(E)** Microbial taxonomic composition of the fecal microbiota showing the top 20 most abundant genera. The bars show the mean relative abundance. The “Others” category includes the grouping of genera with lower relative abundance than the top 20 genera. Significant differences for all analyses were set at *P* < 0.05.

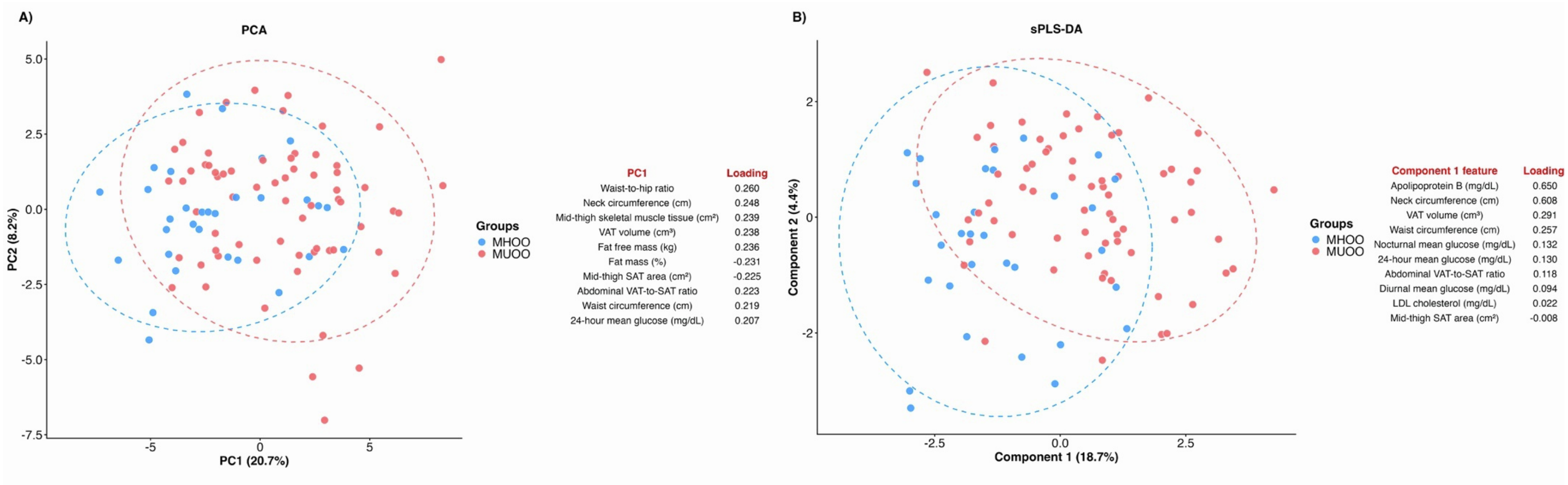

Figure S2. Dimensionality reduction analysis of features differentiating the metabolically healthy overweight/obesity (MHOO) and the metabolically unhealthy overweight/obesity (MUOO) phenotypes in participants from the Study I. (A) Principal component analysis (PCA) and principal component 1 (PC1) features with the ten greatest loading coefficients among 56 outcomes assessed in the Study I cohort (**Table S1**). This analysis, performed in a subsample, included all outcomes used in the pooled analysis of the full sample, as well as adipokines, hepatokines, and alpha diversity indices derived from fecal microbiota. Variables used to define the phenotypes were excluded from this analysis. **(B)** Sparse partial-least-squares discriminant analysis (sPLS-DA) of participants with MHOO and MUOO and loading coefficients of the ten features used to construct component 1. IMAT, intermuscular adipose tissue; LDL, low-density lipoprotein; PC2, principal component 2; SAT, subcutaneous adipose tissue; VAT, visceral adipose tissue.

Table S1. Differences in specific abdominal and mid-thigh adipose tissue depots, and organ-specific fat deposition between participants with metabolically healthy overweight/obesity (MHOO) and metabolically unhealthy overweight/obesity (MUOO), divided by sex.

Men							
	MHOO		MUOO		Difference between groups (MHOO - MUOO)		
	n	Mean (95% CI)	n	Mean (95% CI)	Mean difference (95% CI)	*P* value	Cohen's d
Abdominal adipose tissue depots							
Visceral adipose tissue (cm^3)	38	1407 (1272, 1543)	144	1753 (1659, 1846)	-345 (-513, -178)	**0.002**	0.672
Intermuscular adipose tissue (cm^3)	38	384 (340, 429)	144	411 (384, 438)	-27 (-80, 26)	0.595	0.171
Subcutaneous adipose tissue (cm^3)	38	3035 (2743, 3328)	144	2776 (2631, 2921)	259 (-73, 592)	0.094	0.281
VAT-to-SAT ratio	38	0.51 (0.44, 0.58)	144	0.70 (0.65, 0.75)	-0.19 (-0.28, -0.10)	**< 0.001**	0.680
Mid-thigh adipose tissue depots							
Intermuscular adipose tissue (cm^2)	36	31 (29, 33)	142	33 (32, 35)	-2 (-5, 1)	0.532	0.278
Subcutaneous adipose tissue (cm^2)	36	108 (94, 122)	142	87 (80, 93)	21 (5, 37)	**0.002**	0.494
Organ-specific fat deposition							
Hepatic fat fraction (%)	40	9.0 (7.3, 10.8)	143	12.5 (11.4, 13.7)	-3.6 (-5.9, -1.5)	**< 0.001**	0.536
Pancreatic fat fraction (%)	41	8.7 (6.1, 11.3)	144	8.7 (7.6, 9.8)	0.1 (-2.7, 2.8)	0.595	0.007
**Women**							
	MHOO		MUOO		Difference between groups (MHOO - MUOO)		

	n	Mean (95% CI)	n	Mean (95% CI)	Mean difference (95% CI)	*P* value	Cohen's d
Abdominal adipose tissue depots							
Visceral adipose tissue (cm^3)	54	810 (714, 905)	128	1147 (1072, 1223)	-338 (-461, -215)	**< 0.001**	0.837
Intermuscular adipose tissue (cm^3)	54	289 (258, 321)	128	330 (306, 354)	-41 (-81, -1)	0.146	0.314
Subcutaneous adipose tissue (cm^3)	54	2914 (2701, 3128)	128	3170 (3031, 3308)	-255 (-512, 1)	**0.029**	0.316
VAT-to-SAT ratio	54	0.29 (0.26, 0.33)	128	0.39 (0.36, 0.42)	-0.09 (-0.14, -0.04)	**< 0.001**	0.573
Mid-thigh adipose tissue depots							
Intermuscular adipose tissue (cm^2)	50	25 (23, 26)	121	29 (27, 30)	-4 (-6, -2)	**< 0.001**	0.642
Subcutaneous adipose tissue (cm^2)	50	164 (150, 177)	121	157 (149, 166)	7 (-10, 23)	0.260	0.129
Organ-specific fat deposition							
Hepatic fat fraction (%)	55	5.7 (4.9, 6.4)	130	9.1 (8.1, 10.1)	-3.5 (-4.9, -2.2)	**< 0.001**	0.719
Pancreatic fat fraction (%)	55	4.6 (3.6, 5.7)	131	7.1 (5.8, 8.3)	-2.4 (-4.0, -0.8)	**0.031**	0.419

Data are presented as descriptive means with 95% confidence intervals and as unadjusted raw mean differences (MHOO minus MUOO) with 95% confidence intervals. Two-sided *P* values are derived from adjusted analyses of covariance (parametric or non-parametric, as appropriate) including age and whole-body fat mass as covariates. Therefore, the confidence intervals for raw mean differences are descriptive and are not expected to correspond directly to the reported adjusted *P* values. SAT, subcutaneous adipose tissue; VAT, visceral adipose tissue.

Table S2. Variables included in the principal component analysis (PCA) and sparse partial least squares discriminant analysis (sPLS-DA) for the combined Studies I and II cohorts and for Study I alone.

**Combined Studies I and II cohorts**		**Study I cohort alone**	
**Variable number**	**Variable**	**Variable number**	**Variable**
1	Neck circumference (cm)	1	Neck circumference (cm)
2	Waist circumference (cm)	2	Waist circumference (cm)
3	Hip circumference (cm)	3	Hip circumference (cm)
4	Waist-to-hip ratio	4	Waist-to-hip ratio
5	Fat-free mass (kg)	5	Fat-free mass (kg)
6	Fat mass (kg)	6	Fat mass (kg)
7	Fat mass (%)	7	Fat mass (%)
8	Mid-thigh skeletal muscle tissue (cm^2)	8	Mid-thigh skeletal muscle tissue (cm^2)
9	Fasting insulin (mU/L)	9	Fasting insulin (mU/L)
10	HOMA–IR	10	HOMA–IR
11	HbA1c (%)	11	HbA1c (%)
12	24-hour mean glucose (mg/dL)	12	24-hour mean glucose (mg/dL)
13	Diurnal mean glucose (mg/dL)	13	Diurnal mean glucose (mg/dL)
14	Nocturnal mean glucose (mg/dL)	14	Nocturnal mean glucose (mg/dL)
15	24-hour CV glucose (%)	15	24-hour CV glucose (%)
16	Diurnal CV glucose (%)	16	Diurnal CV glucose (%)
17	Nocturnal CV glucose (%)	17	Nocturnal CV glucose (%)
18	Total cholesterol (mg/dL)	18	Total cholesterol (mg/dL)
19	LDL-C (mg/dL)	19	LDL-C (mg/dL)
20	Apolipoprotein A1 (mg/dL)	20	Apolipoprotein A1 (mg/dL)
21	Apolipoprotein B (mg/dL)	21	Apolipoprotein B (mg/dL)
22	Alanine aminotransferase (U/L)	22	Alanine aminotransferase (U/L)
23	γ–Glutamyltransferase (U/L)	23	γ–Glutamyltransferase (U/L)
24	Alkaline phosphatase (U/L)	24	Alkaline phosphatase (U/L)
25	Sedentary time (min/day)	25	Sedentary time (min/day)
26	LPA (min/day)	26	LPA (min/day)
27	MVPA (min/day)	27	MVPA (min/day)
28	Energy intake (kcal/day)	28	Energy intake (kcal/day)
29	Carbohydrate intake (% EI)	29	Carbohydrate intake (% EI)
30	Fat intake (% EI)	30	Fat intake (% EI)

31	Protein intake (% EI)	31	Protein intake (% EI)
32	Visceral adipose tissue (cm^3)	32	Visceral adipose tissue (cm^3)
33	Abdominal IMAT (cm^3)	33	Abdominal IMAT (cm^3)
34	Abdominal SAT (cm^3)	34	Abdominal SAT (cm3)
35	Abdominal VAT-to-SAT ratio	35	Abdominal VAT-to-SAT ratio
36	Mid-thigh IMAT (cm^2)	36	Mid-thigh IMAT (cm^2)
37	Mid-thigh SAT (cm^2)	37	Mid-thigh SAT (cm^2)
38	Hepatic fat fraction (%)	38	Hepatic fat fraction (%)
39	Pancreatic fat fraction (%)	39	Pancreatic fat fraction (%)
		40	Adiponectin (µg/mL)
		41	DPP4 (µg/mL)
		42	Interleukin–6 (pg/mL)
		43	Leptin (µg/l)
		44	Omentin (ng/mL)
		45	Resistin (ng/mL)
		46	RBP4 (µg/mL)
		47	ANGPTL3 (ng/mL)
		48	ANGPTL4 (ng/mL)
		49	β–Klotho (ng/mL)
		50	Clusterin (µg/mL)
		51	Follistatin (ng/mL)
		52	GDF15 (ng/mL)
		53	SHBG (nmol/L)
		54	Observed features
		55	Shannon entropy
		56	Simpson index

Abbreviations: ANGPTL3, angiopoietin-like 3; ANGPTL4, angiopoietin-like 4; CV, coefficient of variation; DPP4, dipeptidyl peptidase-4; EI, energy intake; GDF15, growth differentiation factor 15; HbA1c, glycated hemoglobin; HOMA–IR, homeostasis model assessment of insulin resistance; IMAT, intermuscular adipose tissue; LDL-C, low–density lipoprotein cholesterol; LPA, light physical activity; MVPA, moderate-to-vigorous physical activity; RBP4, retinol-binding protein 4; SAT, subcutaneous adipose tissue; SHBG, sex hormone-binding globulin; VAT, visceral adipose tissue.
